# The predictive value of respiratory function tests for non-invasive ventilation in amyotrophic lateral sclerosis

**DOI:** 10.1186/s12931-017-0624-8

**Published:** 2017-07-25

**Authors:** T. B. M. Tilanus, J. T. Groothuis, J. M. C. TenBroek-Pastoor, T. B. Feuth, Y. F. Heijdra, J. P. L. Slenders, J. Doorduin, B. G. Van Engelen, M. J. Kampelmacher, J. Raaphorst

**Affiliations:** 1 Department of Neurology, Donders Institute for Brain, Cognition and Behaviour, Radboud University Medical Center, Nijmegen, The Netherlands; 2 Department of Rehabilitation, Donders Centre for Neuroscience Nijmegen, Nijmegen, The Netherlands; 3 Department of Health Evidence, Radboudumc Nijmegen, Nijmegen, The Netherlands; 4 Department of Pulmonary Diseases, Radboudumc Nijmegen, Nijmegen, The Netherlands; 5 Department of Neurology, Academic Medical Centre Amsterdam, Amsterdam, The Netherlands; 6 Home Ventilation Service, University Medical Centre Utrecht, Utrecht, The Netherlands

**Keywords:** ALS, Respiratory function tests, Non-invasive ventilation

## Abstract

**Background:**

Non-invasive ventilation (NIV) improves survival and quality of life in amyotrophic lateral sclerosis (ALS) patients. The timing of referral to a home ventilation service (HVS), which is in part based on respiratory function tests, has shown room for improvement. It is currently unknown which respiratory function test predicts an appropriate timing of the initiation of NIV.

**Methods:**

We analysed, retrospectively, serial data of five respiratory function tests: forced vital capacity (FVC), peak cough flow (PCF), maximum inspiratory and expiratory pressure (MIP and MEP) and sniff nasal inspiratory pressure (SNIP) in patients with ALS. Patients who had had at least one assessment of respiratory function and one visit at the HVS, were included. Our aim was to detect the test with the highest predictive value for the need for elective NIV in the following 3 months. We analysed time curves, currently used cut-off values for referral, and respiratory function test results between ‘NIV indication’ and ‘no-NIV indication’ patients.

**Results:**

One hundred ten patients with ALS were included of whom 87 received an NIV indication; 11.5% had one assessment before receiving an NIV indication, 88.5% had two or more assessments. The NIV indication was based on complaints of hypoventilation and/or proven (nocturnal) hypercapnia. The five respiratory function tests showed a descending trend during disease progression, where SNIP showed the greatest decline within the latest 3 months before NIV indication (mean = −22%). PCF at the time of referral to the HVS significantly discriminated between the groups ‘NIV-indication’ and ‘no NIV-indication yet’ patients at the first HVS visit: 259 (±92) vs. 348 (±137) L/min, *p* = 0.019. PCF and SNIP showed the best predictive characteristics in terms of sensitivity.

**Conclusion:**

SNIP showed the greatest decline prior to NIV indication and PCF significantly differentiated ‘NIV-indication’ from ‘no NIV-indication yet’ patients with ALS. Currently used cut-off values might be adjusted and other respiratory function tests such as SNIP and PCF may become part of routine care in patients with ALS in order to avoid non-timely initiation of (non-invasive) ventilation.

## Background

Amyotrophic lateral sclerosis (ALS) is a fatal neurodegenerative disorder, characterized by progressive muscle weakness, including the respiratory muscles. Respiratory muscle weakness often starts as nocturnal hypoventilation during sleep and symptoms can be treated with non-invasive ventilation (NIV), which improves survival and quality of life in a selection of patients with ALS [1, 2]. The initiation of NIV is partly based on complaints of nocturnal hypoventilation, such as morning headache, daytime sleepiness and loss of concentration or memory. These complaints are not specific and may even be absent, which may lead clinicians to rely, in addition, on daytime tests of respiratory muscle function or pulmonary function [3]. Although new methods are being investigated in ALS (e.g. nocturnal capnography and oxymetry), this is not current practice in most hospitals; the forced vital capacity (FVC) is the most frequently used respiratory function test in patients with ALS [4, 5]. A recent study in the Netherlands showed that 37% of the patients with ALS, at their first visit at a home ventilation service (HVS), already had a level of respiratory impairment that lead to an outright NIV indication, either elective (<3 weeks; 19%), or even in a (sub)acute setting (in 18%) [4].

Ideally, patients with ALS should be referred to an HVS, at least three months before receiving an NIV indication. Then, patients, relatives and physicians can make a considered decision about all possible treatment options [6]. It is not entirely investigated which respiratory function test enables appropriate timing of the referral and indication of NIV in ALS [7, 8]. Compared to the FVC, the sniff nasal inspiratory pressure (SNIP) provides more accurate prognostic information on mortality in patients with ALS, but its value for the prediction of an NIV indication has not been studied in a large ALS cohort [9–13]. An impaired cough is associated with a higher risk for developing respiratory complications. Peak cough flow (PCF) estimates cough efficacy and airway clearance and may help decisions regarding cough augmentation. The role of PCF in predicting the need for NIV is not yet fully clarified [14–16]. Furthermore, the maximum inspiratory and expiratory pressures (MIP and MEP) may have a higher sensitivity for nocturnal hypoventilation compared to the FVC in patients with ALS [17, 18]. We hypothesized that a decline in one of the respiratory function tests, may predict an NIV indication in patients with ALS within the following three months. Therefore, we aimed to examine serial data of FVC, PCF, MIP, MEP and SNIP for the prediction of the need for NIV.

## Methods

### Patients

This study was performed at the Radboud university medical centre (Radboudumc) in Nijmegen and the HVS in Utrecht (2 patients switched to another HVS as they moved places; data of these patients were retrieved from their HVS in Groningen and Maastricht).

Inclusion criteria were a diagnosis of definite ALS, according to the El Escorial criteria [19]. We included patients with progressive muscular atrophy (PMA) because of similarities between PMA and ALS regarding clinical course, genetic background and pathology in the spinal cord [20]. For PMA patients, we used criteria that have been described before: progressive lower motor neuron signs and symptoms in 2 or more of the 4 regions (bulbar, cervical, thoracic and lumbar) with careful exclusion of mimics such as multifocal motor neuropathy, and no clinical upper motor neuron signs and symptoms [21].

In addition, two or more respiratory functions tests (FVC, PCF, MIP, MEP or SNIP) had to be assessed at least at the Radboudumc or HVS and at least one daytime capillary blood gas sample had to be taken at the HVS. We excluded patients with a diagnosis of primary lateral sclerosis (PLS), and ALS and PMA patients with severe bulbar impairment or other, physical or cognitive impairments which made the application of NIV impossible, as judged by the referring or HVS physician.

### Study design, data collection and processing

This is a retrospective cohort study. Case records from 2008 up to 2015 were selected. Retrospectively designed studies do not need approval by the local ethics committee. As all tests had been performed for clinical use, there was no need to obtain informed consent. The following data (anonymized by the main researcher) were retrieved:

1. Respiratory function tests during consecutive outpatients visits: FVC (%predicted), PCF (L/min), MIP (cm H_2_O), MEP (cm H_2_O) and SNIP (cm H_2_O).
2. Data of the first assessment and follow-up visits at the HVS: Daytime capillary PCO_2_ and bicarbonate levels, complaints of respiratory impairment (presence of dyspnoea, orthopnoea, morning headache, fatigue and excessive saliva; yes/no) and indication for NIV initiation (yes/no, as judged by the physician of the HVS)

### Respiratory function tests

All tests were performed in the upright position, using a spirometer (Spirostik; Geratherm Bad Kissingen, Germany), a peak flow meter (MicroPeak™; CareFusion, San Diego, USA) or a respiratory pressure meter (Micro RPM™-120; Micro Medical Limited, Rochester, England). All devices are standardized according to the ATS/ERS Statement [22].

#### Forced vital capacity

FVC is the maximal volume of air exhaled with maximally forced effort from a maximal inspiration. The value is expressed as a percentage of the predicted value, based on patient’s age and height [14].

#### Peak cough flow

PCF is measured by performing a maximal inspiration, followed by a cough as forcefully as possible, while the lips are sealed tightly around the tube [23].

#### Maximum inspiratory and expiratory pressure

MIP and MEP are measured by a non compressible face mask. The MIP is retrieved by exhaling to residual volume and then inhaling with as much effort as possible for at least 3 s. The MEP is retrieved similarly but the opposite way, by inhaling followed by a forced expiration. The maximum value of three manoeuvres is used for MIP and MEP, expressed as cm H_2_O or % predicted value (calculated according to reference values of Wilson et al.) [22, 24, 25].

#### Sniff nasal inspiratory pressure

SNIP is measured by inserting a nose piece or cone into the nostril. The SNIP is retrieved by a maximal sniff starting from relaxed end expiration, performed while the contralateral nostril is closed by an occluding plug. The maximum pressure value of five attempts is used, expressed as cm H_2_O or % predicted value [22, 26].

### Referral process of patients with ALS to home ventilation services

Before referral to an HVS, patients are, on average, trimonthly monitored by one of the multidisciplinary ALS care teams. A referral to an HVS is indicated when one or more of the following occurs: FVC <70%, symptoms of nocturnal hypoventilation, signs of increased breathing activity or daytime hypercapnia (PCO_2_ > 45 mmHg) [27]. Other respiratory parameters, such as PCF or SNIP, are used infrequently in ALS clinics in Netherlands [4]. At the first HVS assessment, capillary blood gas analysis is performed and an extensive medical history is taken, including signs of nocturnal and daytime hypoventilation. Also, patients are informed about the treatment options: non-invasive ventilation (NIV); invasive ventilation by tracheostomy (TPPV) or palliative care. If a patient opts for ventilatory support in the future, his or her respiratory function will be measured trimonthly at the HVS using the FVC, PCF, MIP, MEP and SNIP to monitor disease progression. Elective NIV indications are made by HVS physicians only. The NIV indication is based on either proven nocturnal or daytime hypercapnia, orthopnoea and/or other complaints of nocturnal or daytime hypoventilation [9, 10].

### Data and statistical analysis

Patients’ characteristics are reported as proportions and mean ± standard deviation (SD). A time-curve was used to evaluate the decline of the respiratory function tests of patients with two or more consecutive measurements before receiving an NIV indication. Differences in respiratory function tests between 6 months and 3 months before NIV indication and in the latest 3 months were assessed by paired t-tests. To explore the test results of patients who received an NIV indication, the value of the FVC, PCF, MIP, MEP and SNIP was plotted against the cumulative percent of patients with an NIV indication within the following 3 months. Based on these data, tentative cut-off values were determined at an arbitrary value of 85%, with a 95% confidence interval (the value of 15% is a reduction of more than half compared to the proportion of 37% in our previous study and is thought to reflect a clinical relevant difference) [4]. Sensitivity and specificity of these cut-off values, and cut-off values of international guidelines were determined by ROC-curves with the values of *t* = −3 as positive outcome and values of *t* = −6 and *t* = −9 as negative outcome.

Respiratory function test results at the last visit at the ALS clinic of the Radboudumc were compared between two groups, stratified by NIV indication at the first visit at the HVS, using an independent samples t-test, not assuming equal variances (Satterthwaite method).

All statistical analysis were done using IBM SPSS statistics version 23. A two-tailed *p*-value of <0.05 was considered significant.

## Results

**Fig. 1:**
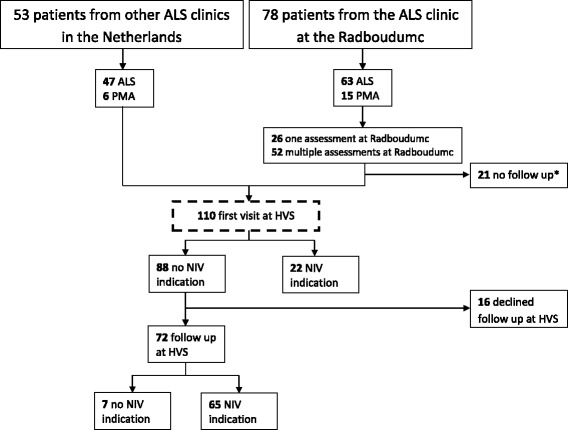
Flowchart of the patients. The *dotted box* indicates the patients included for analyses. *21 patients who did not yet receive a referral to an Home Ventilation Services (HVS) or died before. NIV: Non-invasive ventilation (successfully accepted (*n* = 77) use >48 h), ALS: Amyotrophic Lateral Sclerosis, PMA: Progressive Muscular Atrophy

Medical records of 131 patients were examined (Fig. 1). 21 patients did not visit an HVS and were excluded from this study. Table 1 shows the characteristics of the 110 included patients. Fifty-seven patients (52%) were referred to an HVS by the multidisciplinary ALS care team at the Radboudumc. From 53 other patients, referred by other ALS care teams, data were retrieved at the HVS in Utrecht; no data concerning measurements at their ALS care team were available.

**Table 1:** Characteristics of the included patients

	All patients (*n* = 110)
Demographic and disease variables	
Male (%)	60.3
Diagnosis ALS/PMA (% ALS)	84.0
Onset spinal/bulbar/both (%)	60.3 / 23.7 / 16.0
Age at referral (years)	61.5 ± 12.0
Disease duration (years)°	2.0 ± 1.7
Mortality (n)°	67
Variables at first evaluation at the HVS	
Interval referral and first HVS assessment (weeks)	5.6 ± 5.1
PCO2 (mmHg)	39.4 ± 5.6
Bicarbonate (mmol/L)	26.40 ± 3.69
Follow up until NIV (months)*	9 ± 8

Values are mean (SD) unless stated otherwise. Time of death was checked in the Municipal Personal Database at January 1th, 2016 [38]. °Missing data of 20 patients, *n* = 90. *Only patients who received an NIV indication, *n* = 87. *ALS* Amyotrophic lateral sclerosis, *PMA* Progressive Muscular Atrophy, *HVS* Home ventilation services, *NIV* Non-invasive ventilation

Out of the 110 patients, 87 patients (79%) received an NIV indication at the HVS; the remaining 23 patients did not receive an NIV indication yet or died before receiving one. 12%, 21%, 28% and 40% of the patients had 1, 2, 3 or 4 or more assessments of respiratory function tests before the NIV indication, respectively. All patients had had at least one blood gas analysis at the HVS. After receiving an NIV indication, 77 patients (89%) successfully accepted NIV more than 48 h. Out of the 77 patients, 4 patients chose to be ventilated by tracheostomy when NIV was insufficient.

### Decline in respiratory function

**Fig. 2:**
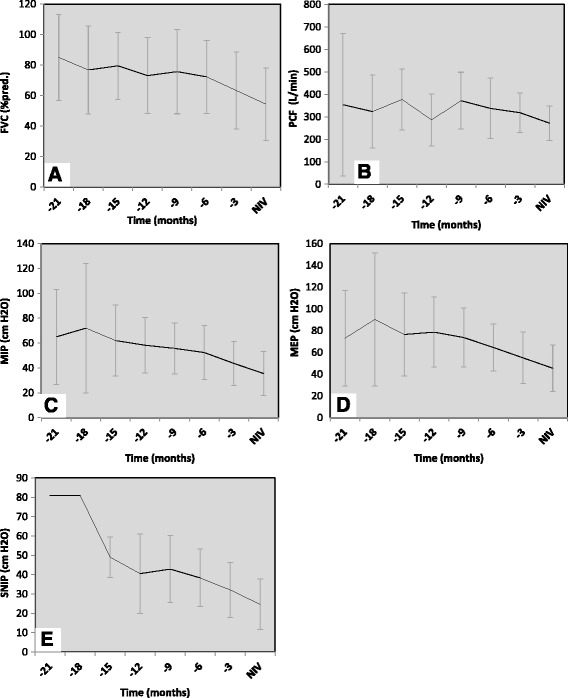
Time-curves of patients with 2 or more consecutive measurements**. a** FVC: Forced Vital Capacity (% predicted value), *n* = 2, 3, 9, 17, 28, 43, 67, 67; respectively. **b** PCF: Peak Cough Flow (L/min), *n* = 2, 4, 8, 10, 12, 21, 24, 24; respectively. **c** MIP: Maximum Inspiratory Pressure (cm H_2_O), *n* = 2, 3, 9, 15, 24, 38, 64, 64; respectively. e MEP: Maximum Expiratory Pressure (cm H_2_O), *n* = 2, 3, 8, 15, 24, 38, 64, 64; respectively. **e** SNIP: Sniff Nasal Inspiratory Pressure (cm H_2_O), *n* = 1, 1, 3, 3, 7, 12, 31, 31; respectively. Data are presented as mean (SD) and time intervals are presented in months before the indication of non-invasive ventilation (NIV)

All five respiratory function tests showed a descending trend between the trimonthly assessments. Average time curves are displayed in Fig. 2a-e. The mean declines within the last 3 months before the NIV indication were 16% ±21(FVC, *n* = 67), 16% ±19 (PCF, *n* = 17), 18% ±30 (MIP, *n* = 64), 13% ±34(MEP, *n* = 64) and 22% ±28 (SNIP, *n* = 31), respectively; the difference between *t* = −3 months and t = NIV indication was significant for all tests (*p* < 0.001). The difference between *t* = −6 months and *t* = −3 months was 16% (FVC; *p* < 0.001, *n* = 44), 2% (PCF; *p* = 0.219, *n* = 13), 18% (MIP; *p* < 0.001, *n* = 43), 17% (MEP; *p* < 0.001, *n* = 42) and 15% (SNIP; *p* < 0.007, *n* = 15), respectively.

### Cut off values

**Fig. 3:**
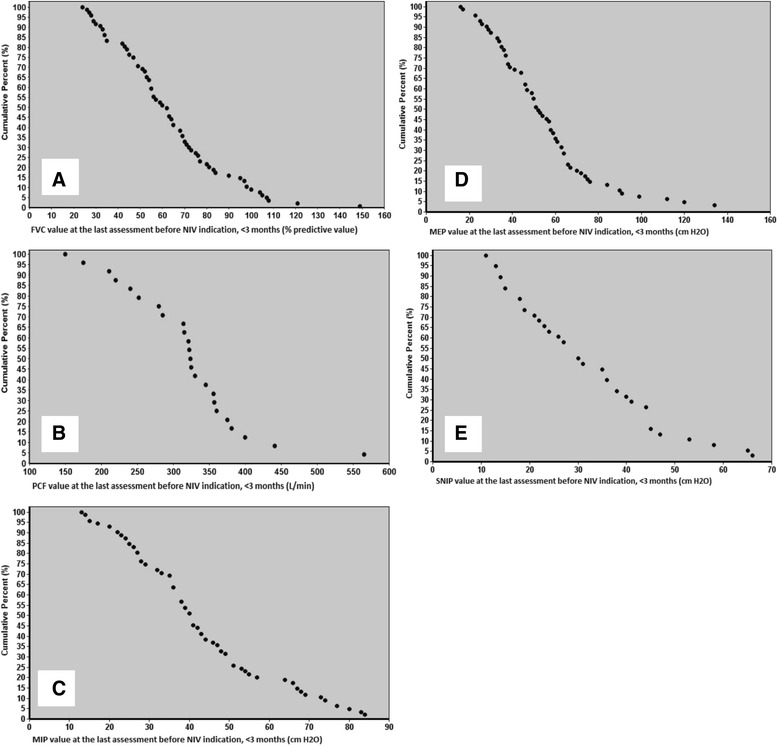
Respiratory function tests plotted against the proportion of patients with an NIV indication within the following 3 months**. a** FVC: Forced Vital Capacity (% predicted value), *n* = 72; **b** PCF: Peak Cough Flow (L/min), *n* = 24; **c** MIP: Maximum Inspiratory Pressure (cm H_2_O), *n* = 70; **d** MEP: Maximum Expiratory Pressure (cm H_2_O), *n* = 70; **e** SNIP: Sniff Nasal Inspiratory Pressure (cm H_2_O), *n* = 38. NIV: Non-invasive ventilation. For example, 15% of the patients had a PCF of 386 L/min or more in the 3 months before their NIV indication

**Fig. 4:**
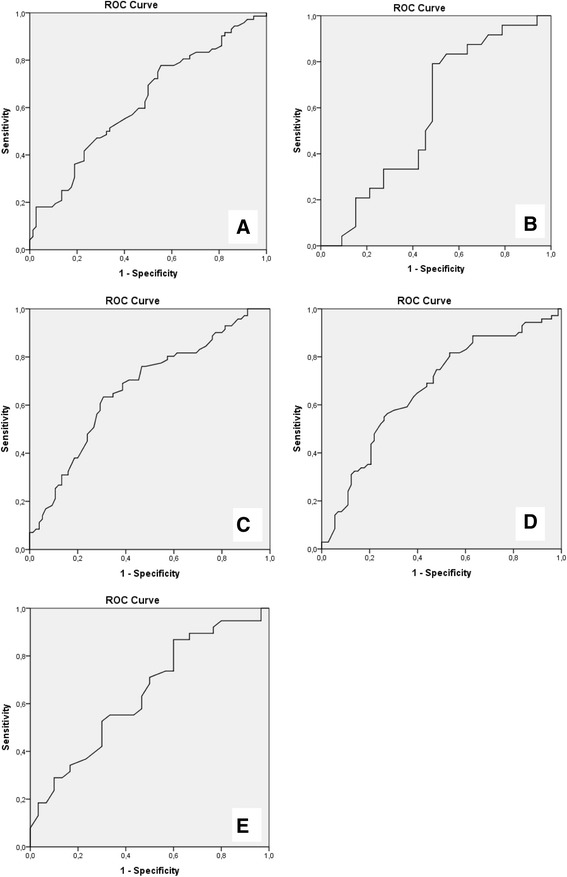
ROC curves**. a** FVC: Forced Vital Capacity (% predicted value); **b** PCF: Peak Cough Flow (L/min); **c** MIP: Maximum Inspiratory Pressure (cm H_2_O); **d** MEP: Maximum Expiratory Pressure (cm H_2_O); **e** SNIP: Sniff Nasal Inspiratory Pressure (cm H_2_O). The graphs are calculated with the values of the measurements of *t* = −3 as positive outcome and of *t* = −6 and *t* = −9 as negative outcome

Figure 3a-e shows values of respiratory function tests against the cumulative percentage of patients with an NIV indication within the following 3 months. Cut off values were determined at a value where 85% of the patients received an NIV indication within 3 months, shown in Table 2. Sensitivity and specificity were calculated for these cut-off values (Table 2). In addition, sensitivity and specificity of previously published cut-off values were determined. The ROC curves are shown in Fig. 4a-e.

**Table 2:** Cut-off values for referral to the home ventilation services

	Suggested values			Guideline values [9, 15, 27]		
	Cut-off value	Sensitivity	Specificity	Cut-off value	Sensitivity	Specificity
FVC	95 (77–104)	85	22	70	65	50
PCF	386 (356–472)	88	36	300	33	67
MIP	67 (52–74)	85	27	60	82	35
MEP	74 (66–90)	85	38	60	68	56
SNIP	45 (41–59)	87	40	40	71	50

Cut-off values are presented as value (95% CI). The sensitivity and specificity are calculated with an ROC curve analysis. *ALS* Amyotrophic Lateral Sclerosis, *HVS* Home Ventilation Services, *NIV* Non-invasive ventilation, *FVC* Forced Vital Capacity (% predicted value), *PCF* Peak Cough Flow (L/min.), *MIP* and *MEP* Maximum Inspiratory and Expiratory Pressure (cm H_2_O), *SNIP* Sniff Nasal Inspiratory Pressure (cm H_2_O)

### Respiratory function tests at the first HVS assessment

Out of the 57 patients of the Radboudumc (52% of the 110 included patients), 22 patients received an NIV indication at their first assessment at the HVS and were classified as ‘NIV indication’; 35 other patients were classified as ‘no NIV indication yet’. Table 3 shows the characteristics of these groups. The mean age of the ‘NIV indication’ group was higher (69.8 ± 10.3 years), than the ‘no NIV indication yet’ group (61.4 ± 13.1 years), *p* = 0.014. In Table 4, the respiratory function tests and complaints (at time of referral) are displayed for groups stratified by NIV indication at the first HVS visit. The time between the last assessment at the ALS clinic and the first visit at the HVS was 6 ± 5 weeks and showed a trend towards a shorter interval in the patients who received an NIV indication compared to the ‘no NIV indication yet’ group (4 ± 3) vs. 7 ± 6; *p* = 0.056). The mean (SD) PCF was lower in the ‘NIV indication’ group compared to the ‘no NIV indication yet’ group (259 ± 92 L/min vs. 348 ± 135 L/min, *p* = 0.019). Complaints of nocturnal hypoventilation (assessed at the ALS care team at the Radboudumc) tended to be more frequent in patients with an NIV indication (17 out of 22), compared to the group with no NIV indication yet (18 out of 35), *p* = 0.051.

**Table 3:** Characteristics of the patients of the Radboudumc

	All patients (*n* = 57)	No NIV indication (*n* = 35, 61.4%)	NIV indication (*n* = 22, 38.6%)	*P*-value
Demographic and disease variables				
Male (%)	50.9	54.3	45.5	0.516
Diagnosis ALS/PMA (% ALS)	87.7	88.6	86.4	0.805
Onset Spinal/Bulbar/Both (%)	45.6 / 36.8 / 17.5	40.0 / 37.1 / 22.9	54.5 / 36.4 / 9.1	0.352
Age at referral (years)	64.6 ± 12.7	61.4 ± 13.1	69.8 ± 10.3	0.014
Disease duration (years)	1.9 ± 1.6	2.0 ± 1.6	1.8 ± 1.8	0.572
Deceased (n)	34	19	15	0.298
Variables at HVS				
Interval referral and first assessment (weeks)	5.6 ± 5.1	6.6 ± 5.9	4.1 ± 2.9	0.056
pCO2 at first evaluation (mmHg)	40.6 ± 6.3	37.92 ± 3.19	44.95 ± 7.70	<0.0001
Bicarbonate at first evaluation (mmol/L)	26.7 ± 3.7	25.08 ± 2.51	29.23 ± 3.88	<0.0001
Follow up until NIV (months)*	12 ± 6	12 ± 6	0 ± 0	-

The groups are based on the presence of an NIV indication during the first assessment at the HVS. Values are mean ± SD unless stated otherwise. *P*-values: comparison between patients with and without an NIV indication at the first visit at the HVS, using t-tests and Pearson Chi-square. *ALS* Amyotrophic Lateral Sclerosis, *PMA* Progressive Muscular Atrophy, *HVS* Home Ventilation Services, *NIV* Non-invasive ventilation. *Only patients who received an NIV indication

**Table 4:** Comparison of respiratory function tests and complaints at referral, between patients with and without an NIV indication at the first home ventilation service visit

	No NIV indication yet (*n* = 35)		NIV indication (*n* = 22)			
	N		N		Difference (SE)	*P*-value
FVC	32	79 ± 21	20	70 ± 20	−9.9 ± 6	0.101
PCF	24	348 ± 137	16	259 ± 92	−88.4 ± 39	0.019
MIP	28	59 ± 36	19	50 ± 26	−8.9 ± 9	0.338
MEP	29	46 ± 23	18	44 ± 21	−1.9 ± 7	0.774
SNIP	15	40 ± 18	5	28 ± 9	−12 ± 12	0.089
Complaints	35	51%	22	77%		0.051

Data are presented as mean ± SD or proportion. These data are collected at the Radboudumc at the time of referral to the HVS. The NIV indication was made at the first visit at the HVS. Median time between referral and the first visit at the HVS was 5.6 weeks (range 4.4–7.0). *HVS* Home Ventilation Services, *NIV* Non-invasive ventilation, *FVC* Forced Vital Capacity (% predicted value), *PCF* Peak Cough Flow (L/min.), *MIP and MEP* Maximum Inspiratory and Expiratory Pressure (% predicted value), *SNIP* Sniff Nasal Inspiratory Pressure (% predicted value)

## Discussion

The present study examined serial data (trimonthly) of five respiratory function tests (FVC, PCF, MIP, MEP and SNIP) in patients with ALS in order to determine their predictive value for NIV indication. The main findings of this study are: (1) PCF has the largest predictive value for NIV indication, with a significant discrimination between ‘NIV indication’ patients and ‘no NIV indication yet’ patients at their first HVS visit; (2) all of the tests showed a descending trend with a significant difference in the last months before the NIV indication (*p* < 0.001), and SNIP showed the greatest decline. Below we discuss our findings according to three tests: FVC; PCF and SNIP.

### Forced vital capacity

Supine FVC has shown the ability to detect diaphragmatic weakness [28] and guidelines suggest that referral to an HVS is indicated when FVC is <70% of the predicted value [27]. Previous cross-sectional studies however, have demonstrated the limitations of the FVC in terms of detection of early respiratory dysfunction (and as such as a criterion for timely referral to an HVS) [4, 11, 12]. One could assume that by using longitudinal data, the FVC may have a better predictive value, however the present retrospective study corroborates the restricted value of FVC to detect imminent respiratory insufficiency, even if serial data are used. Data of the current study show that when using an FVC <70% of the predicted value, 35% of the patients are inadvertently not referred to an HVS within the following 3 months, while they could have benefitted from such a referral [4]. This proportion is comparable to our previous study [4] and are in line with a recent study of Polkey et al., who investigated the predictive value of invasive and non-invasive respiratory muscle strength assessments for survival or ventilator-free survival. The latter study showed a good predictive power of FVC for ventilation-free survival, but the cut off value indicating a poor prognosis, lies within the normal or near normal range (>80% predicted) [29].

### Peak cough flow

As respiratory muscle weakness leads to impaired cough with a lower PCF, we hypothesized that a decline of the PCF may predict the need for NIV in the following months [14]. Indeed, time curves of serial PCF measurements showed a decline of 16% before receiving an NIV indication in the last 3 months. A similar percentage has been found by others who used peak expiratory flow time (PEFT). PEFT increased significantly (indicating more impairment) and linearly with time, 4.7% per month (*p* < 0.001), which is comparable to our findings (PCF −16% in 3 months = −5.3% per month) [30, 31]. Polkey et al. also showed a descending trend of the peak cough pressure in the months previous to NIV or death [29]. Our data further showed that the PCF, as measured 5.6 weeks before the first HVS assessment, was the only test that discriminated ‘NIV indication’ from ‘no NIV indication yet’ patients. A predictive value of PCF for the need for NIV has been found previously in patients with ALS who had an acute lower respiratory tract infection [16]. Together, our findings and previous findings by others suggest the value of PCF, which should lead to more frequent use of this measure during the respiratory care of patients with ALS [29–31]. A national survey among referring physicians in the Netherlands suggests room for improvement: PCF was routinely used by half of Dutch ALS physicians (paper in preparation). In a comparable national survey in the UK, the PCF was not included [5].

Patients with an NIV indication were older at the time of referral than the ‘no NIV indication yet’ group. In normal subjects, PCF is generally >500 L/min for all ages [32]. Previous studies showed an (inverse) age effect of the Maximum Expiratory Flow (MEF) and Peak Expiratory Flow (PEF), which was, however, limited when disease effects were excluded [33, 34]. The effect of age on the PCF has been shown in three age groups: 8–15 years, 16–35 years and >36 years; however, we are not aware of an important age effect at ≥60 years [34]. Although we cannot exclude a small effect, it is unlikely that the PCF difference of 89 L/min is due to an age difference.

### Sniff nasal inspiratory pressure

The difference of the SNIP value between ‘NIV indication’ and ‘no NIV indication yet’ patients was considerable: 28 vs. 40% of the predicted value, although not statistically significant. The latter is probably related to the relatively low number of patients with (serial) SNIP measurements (*n* = 20 in our study), in particular when we consider previous findings of the SNIP being able to detect hypercapnia, nocturnal hypoventilation or survival in ALS [11, 29, 35, 36]. The significant descent of SNIP values in the months before receiving NIV in our study and (prior to NIV or death) in another study (*n* = 78) corroborates the importance of SNIP measurements in the clinical assessment of patients with ALS and possible deterioration of respiratory muscle strength [29].

Similarly to the PCF, the SNIP is performed routinely by only a limited number of physicians in the Netherlands (13%; paper in preparation). In the UK 4% of the physicians used the SNIP routinely and 13% of the physicians used SNIP only if patients were symptomatic [5].

### Timing and site of onset

The cut-off values that were determined resulted in a high sensitivity to predict an NIV indication within the following 3 months, however specificity was low. This implies that the number of false positive referrals to an HVS is relatively high. However, in our opinion, an early referral is preferable compared to a late referral considering the importance for patients with ALS to make a decision about the possible treatment options. Costs and burden of non invasive respiratory function tests are minimal and compensates the benefit of a timely or even early start of NIV: it has proven to postpone the decrease of respiratory muscle strength and thereby prolong survival [37]. Although bulbar onset can be challenging for NIV to be effective, a recent survey among Dutch referring physicians showed that only 16% stated bulbar impairment as a reason to refrain from referral (paper in preparation). Current practice, at least in the Netherlands, does not exclude these patients form referral to an HVS.

In addition to the strengths of this study (analysis of serial measurements and combining data of an ALS care team and an HVS), one of the limitations is the retrospective design, which is the main reason for the missing values of respiratory function measurements.

## Conclusion

Out of five respiratory function tests, PCF and SNIP have the best predictive value for an NIV indication in patients with ALS. We therefore recommend to use PCF and SNIP more often during the follow-up of patients with ALS. Our findings and proposed cut offs, may serve as a starting point for future research, aimed at optimal referral of patients with ALS to an HVS, in order to avoid emergency initiation of NIV as much as possible [6].

## Abbreviations

ALS: Amyotrophic Lateral Sclerosis
FVC: Forced Vital Capacity
HVS: Home Ventilation Service
MEF: Maximum Expiratory Flow
MEP: Maximum Expiratory Pressure
MIP: Maximum Inspiratory Pressure
NIV: Non-Invasive Ventilation
PCF: Peak Cough Flow
PEFT: Peak Expiratory Flow Time
PLS: Primary Lateral Sclerosis
PMA: Progressive Muscular Atrophy
SNIP: Sniff Nasal Inspiratory Pressure
TPPV: Invasive ventilation by tracheostomy
